# Retraction Note: Lessons from the Winter Paralympic Games disclosing the epidemiology of winter sports injury in paralytic athletes: a meta-analysis

**DOI:** 10.1186/s13102-024-00994-3

**Published:** 2024-09-26

**Authors:** Fengyu Wu, Yitong Liu, Maohua Zhuang

**Affiliations:** 1 School of Sport Science, Harbin University of Physical Education, Harbin, 150008 China; 2 Graduate School, Harbin University of Physical Education, Harbin, 150008 China; 3 Physical Education and Training, Harbin University of Physical Education, No. 1 Dacheng Street, Nangang District, Harbin, 150008 China

**Retraction Note: BMC Sports Science, Medicine and Rehabilitation (2022) 14:53**

10.1186/s13102-022-00446-w

The Editor has retracted this article. After publication, concerns were raised regarding the statistical analysis in the article. Specifically, it is stated that the authors used the “Begger’s test”, which is not described in the literature. The Editor therefore no longer has confidence in the presented data.

None of the authors have responded to any correspondence from the editor or publisher about this retraction.

